# Syphilinum

**Published:** 1891-09

**Authors:** Thomas Wildes

**Affiliations:** Kingston, Jamaica


### SYPHILINUM.

THOMAS WILDES, M. D., KINGSTON, JAMAICA.

(Concluded from page 275.)

About one year ago a boy of four and a half years was brought to me for an obstinate eruption on his face which was apparently of a syphilitic origin, but which generally elsewhere was still more prominently a combination of prurigo and herpes, each being separately distinguishable. The rash was on his chin, lips, cheek-bones, forehead, and hairy scalp; on his arms, chest, back, in the bends of and on the joints, and on his feet and hands—not in any great quantity, and nowhere profuse. (We are usually taught that herpes and syphilis are not related. I am convinced that the contrary is the fact. According to Hebra prurigo is of *unknown origin*, and is not related to syphilis. He also claims that it is met with only among the lower classes. I can show him that prurigo is one of the initial stages of leprosy, that it is transmissible, and that it is met with among all classes, rich and poor alike. Moreover that it is cured with Syphilinum, and I have so cured it, although we are told that "syphilis never itches." Hebra says that prurigo is never contagious. I announce it as decidedly infectious. He says it is incurable. I cure it. I find always that pathologists are *weak reeds* for the sick to lean upon.) This boy had a spot of eruption on his left thigh the size of the first joint of my thumb. It was about six inches below the great trochanter and back of the femur line. This piece of eruption was distinctly a leper spot. The *tout ensemble* in this boy made a precious combination. I began treatment by exhibiting the recognized remedies for herpes and prurigo. For four months I made no progress, save that the general character of the eruption got better and worse. I was then just beginning to confirm my belief in the curability of leprosy, and of its close relation to hydra-headed syphilis. I gave this boy Syphilinum$^{m}$, Swan, and ordered a dose every night. Shortly afterward the mother brought him again, stating that he was much worse. The rash was out strongly all over his body, in patches, and his face was one-third covered with a thick, yellow, scabby eruption, having a fiery base, and containing a watery, gummy, yellow scum. I reassured the mother, and had her continue the remedy. To all appearances the boy is now well, and his general health has improved wonderfully. He is no longer nervous, is growing nicely, has good appetite, is strong, sleeps well; all of which he was heretofore behind in. His father and mother are quadroons, and are apparently carrying about with them the usual negro constitutional syphilis and leprosy mixed, in latent form. He is an only child. His cousin, aged ten, was long under my care for an obstinate laryngitis.

A boy of twenty months, fourth child of the mother, was brought to me for the following symptoms one week ago—to wit: Fretful, peevish, cross and crying, tossing in his sleep, grinding his teeth, face dotted here and there with little papules, filled with a watery yellowish matter, most on edges of eyelids where they were largest, and as one would disappear another would come, teeth irregular, and unlike those of the three older children, arms and legs emaciated, very tottery on his feet, and often stumbling and falling when trying to walk. Very nervous. Mother thought he had worms. I said "No, these worm symptoms come always from two causes; either *reflex*, from worms in the bowels causing passive conjestion of base of brain, or *direct* from constitutional causes producing a similiar though more durable congestion."

Having known the husband for over three years, I questioned the mother cautiously and carefully. Could get no history of syphilis. Nevertheless, I gave Syphilinum$^{m}$, Swan, and ordered a dose every night. The next day he was brought to me with violent earache, which had kept the whole house awake from two A. M. I ordered Glycerine and hot Olive oil mixed, and dropped in the ear, not hot enough to burn him. Have seen child every day. The pains were quickly allayed, the ear has discharged freely and now the papulous eruption is disappearing and the skin is assuming a dirty, cachetic hue, the eyes discharge a watery, gummy substance, causing the lids to adhere, especially at night, and in every other way the child is much better. The father denies syphilis, but I am convinced that he conceals the fact. He is now almost bald, whereas he had a luxuriant head of hair three years ago. The mother has ulceration of the os uteri, and recently miscarried.

I am now, January, 1891, curing with Syph.$^{m}$, Swan, a young girl æt. sixteen years, of Half-way Tree, this island, whose mother brought her to me on November 6th, 1890. Her history was: Had measles one year ago that did not come out properly. For one and a half years prior thereto she was subject to neuralgic headaches. "Has been very ailing for about two years," to wit: Very despondent, wants to die, and headaches growing more violent. During the headaches the *temple veins stand out*, she has pains all over the body, is very *irritable, excited*, restless, *walking much of the time*, does not wish to be soothed, *violent on being opposed*, has tremors and *seems on the verge of convulsions*, seems dazed, *absent-minded*, and almost *insane. Always washing her hands.* Was formerly very constipated, but now subject to "a kind of diarrhœa." *Menses never have come on properly*, and for past year have been very irregular, much delayed, scanty, and *always extremely painful.* Often feverish. *Sleep, anxious, distressed*, and often *wakeful* and violently *restless.*

Syphilinum covered the above symptoms, as I have learned partly from Dr. Swan, but largely from clinical experience. Moreover, she had one variety of Hutchinson's syphilitic teeth, whereas her mother's teeth were the large, full, rounded, prominent, *psoric* variety, as claimed by Dr. Wildes. Her father was dead, but the history, and also the mother's leucorrhœa seemed to point to his having had syphilis.

Her menstrual pains were those of Nitric acid, Belladonna, Platinum, Pulsatilla, Cocculus, Colocynth, Chamomilla, and Cimicifuga combined. I had not time nor inclination to TRY them all, so gave Syph.$^{m}$, Swan, once per day. She is now almost in perfect health, and surely recovering.

The case of a young lady named in my article on *Leprosy-Syphilis-Vaccination*, who, with her sisters, had contracted lepra-syphilis from vaccination, and whose mother had contracted it by ricochet, was this: She had an immense blood boil on her arm, which would not heal; had been lanced, looked very angry, and had baffled the skill of leading luminaries of the Island to heal. Her face was also sadly broken out, with a lumpy, fiery rash. I handed her a one-drachm vial of alcoholic solution of Syphilinum$^{m}$, Swan, to touch to the tongue (invert the bottle) every night at bedtime. Her recovery was remarkable and rapid, her arm healed quickly, and her face is now free of eruption.

Her mother, to save expense of consultation, also took the medicine on her own authority. She also rapidly recovered from an immense lepra-syphilis blood boil on her neck, which had been very obstinate. The youngest sister, who is my patient-in-chief in that family, also named in the above article, is steadily and rapidly being restored to health, and Syphilinum$^{m}$, Swan, is her sheet-anchor.

In 1882 I cured a Hunterian chancre with Syphilinum$^{m}$, Swan, in a young man suffering from static pneumonia, and at that time a consumptive subject, which had attacked the frænum, and after eating under it and making a cavern, with the frænum for an *arc de triomphe*, finally ate through it, causing a profuse hemorrhage. The recovery was complete, no secondary or tertiary symptoms have appeared up to date, and his health has since been greatly improved.

In 1883 I cured with Syphilinum$^{m}$, Swan, a boy three years old who had clusters of yellow blisters on his fingers and at the roots of the nails, distorting the nails like tetter or herpes would do. He was the son of a lawyer and legislator of South Carolina, who was stricken with epilepsy, followed by some aphasia symptoms after his marriage to his second wife, the boy's mother. He subsequently had recurrent attacks of epilepsy, which precludes the possibility of his having had an attack of aphasia alone. He came to me for an opinion only—was never under my care. He preferred Surgeon Generals, big fees, and Bromide of Potash. The boy was helped with Fluoric acid, cured with Syphilinum, and the nails became straight.

In 1883 a sickly girl of nine years, youngest child of a family that I attended, was brought to me for an attack of conjunctivitis phlyctenularis, involving nearly the entire periphery of the cornea. I could make but little headway beyond allaying the violence of the acute symptoms, and was feeling discouraged, when interstitial keratitis appeared. Then I gave Syphilinum$^{m}$, *Swan*, a dose every night. The child's health improved, the entire cornea soon cleared up, and simultaneously the brow, cheek-bone, and side of the nose broke out in fiery, scabby, syphilitic eczema, elsewhere described in this paper. The mother, becoming impatient at my tardiness in curing the rash, asked and obtained my consent to take the child to some one else who would use salves on it. Quickly the rash disappeared, but shortly the child was brought to me again for keratitis phlyctenularis. Again I cured the eye and drove the rash out, and again the mother levanted in search of the salve treatment. I then refused to have anything more to do with the case, and it ended in my ceasing to treat the family, for another physician was soon installed in their affections.

In 1880 Syphilinum$^{m}$, *Swan*, at once stopped the pain, and eventually cured, an osteo-sarcoma in the centre front of the right tibia of a married man who confessed to a former syphilis. His wife, a beautiful woman, always miscarried and had a horrible leucorrhœa, but never came fully under my care. He had suffered from this growth for three years, it was increasing and had reached the size of half an ostrich egg, and the pains at night were agonizing. It was an irregular, spongy growth of bone, partly laminated and very hard; but the fact that it finally disappeared entirely caused me to differentiate against a true exostosis.

Three years ago, and one and a half years ago, I relieved with Syphilinum$^{m}$, *Swan*, two cases of angina pectoris here in Jamaica. One case had also ptosis of left eye, and facial paralysis, left side, and slight aphasia, all of old standing. For eight years he had been wholly impotent. He was cured of all of these, and was greatly relieved, getting well of his angina; but, like the other case, he left me before I could pronounce the angina cured.

Syphilinnm causes a seething feeling as of hot water or hot oil running through all of the veins of the body, all night long, after taking the first dose, in cases of old standing acquired syphilis. In one such case, an Englishman named Miller, where I had given it for headache, syphilitic paralysis soon followed, with aphasia, imbecility, and incontinence of feces and urine, for which he was sent to Ward's Island Hospital in 1876. After partial recovery, he came out, when I finished the cure and sent him home to England.

Since commencing this paper, a former captain of the British Navy inadvertently confessed to me in conversation that he had syphilis and a bad gonorrhœa combined, many years ago, before he was married. Nearly three years ago I treated and cured ot cholera infantum, his grandchild, after an allopath had failed to do anything but *check* the bowels with chalk mixture, Bismuth, etc. At intervals relapses followed, the third nearly fatal, and then I was called. It was a most tedious and anxious case. I could get no history of syphilis, but the child's father bore traces of hereditary syphilis. Finally, when the case seemed almost hopeless, I began giving Syphilinum$^{m}$, *Swan*, a dose once a day. The child began to recover, when a cold abscess size of a hen's egg developed at the junction of the third and fourth lumbar vertebræ. At first I was misled into believing it to be a *spina bifida*. This was lanced, and Silicea$^{30}$ finished a very pretty cure. The girl is living yet, a very bright child. I could scarcely refrain from hugging the captain when he so innocently confirmed my judgment of nearly three years before! But I succeeded in maintaining a discreet silence.

Many persons, after taking Syphilinum for a few days, complain of heavy, crushing, cutting pain across the base of cerebellum. Others, of heavy aching and stiffness, from base of neck up through muscles and cords of neck, and into the brain. Others, of a heavy, clouded, dull feeling in base of brain, with physical lethargy, and sometimes with dizziness, sometimes with confusion of thoughts, and often a feeling as if one is going insane, or about to be paralyzed. Sometimes a far-away feeling, with apathy and indifference to the future. Accompanying these may come a heavy, dragging, dull feeling in the lumbar region, with stiffness, and want of elasticity.

Almost invariably, in Jamaica, when the patient fails to properly improve from chronic or sub-acute ailments under the appropriate homœopathic remedy, and where I can only know, *inferentially*, from some of the symptoms, that syphilis is present in the blood, I continue the homœopathic remedy, or not, according to the nature of the case, and give Syphilinum$^{m}$, *Swan*, a dose every night. The result is laughable! The patient either gets violently worse, or rapidly better, at once. I count the cure as dating therefrom, and am called a benefactor. Ergo: *Jamaica* and *syphilis* are synonymous terms.

In all cases of acute pains, from iritis, neuralgia, sciatica, rheumatism, periostitis, and such, where the pains are *worse at*

*night*, I invariably give a dose of Syphilinum at bed-time, often thus soothing the pain and usually bringing sleep, and accomplishing within twenty-four hours what other doctors have failed to do for days and even weeks.

I should add, in parenthesis, that for many years I have used *Psorinum*$^{m}$, one dose every night, in all obstinate and seemingly incorrigible cases of pleuro-pneumonia, pleurisy, or peritonitis, where syphilis is not manifest, and where the appropriate remedies, and even a dose of Sulphur at night, could not soothe nor quiet the patient, stop the pain, nor bring sleep. The effect of Psorinum is always marvelous, dating from the first night, and the patient begins to recover.

KINGSTON, JAMAICA, January 21st, 1891.

